# Stroma-derived IL-6, G-CSF and Activin-A mediated dedifferentiation of lung carcinoma cells into cancer stem cells

**DOI:** 10.1038/s41598-018-29947-w

**Published:** 2018-08-01

**Authors:** Carlos F. D. Rodrigues, Eurico Serrano, Maria I. Patrício, Mariana M. Val, Patrícia Albuquerque, João Fonseca, Célia M. F. Gomes, Antero J. Abrunhosa, Artur Paiva, Lina Carvalho, M. Filomena Botelho, Luís Almeida, Isabel M. Carreira, Maria Carmen Alpoim

**Affiliations:** 1 Center for Neuroscience and Cell Biology (CNC), University of Coimbra, 3004-517 Coimbra, Portugal; 2 Center of Investigation in Environment, Genetics and Oncobiology (CIMAGO), Faculty of Medicine, University of Coimbra, 3001-301 Coimbra, Portugal; 3 Faculty of Medicine (FMUC), University of Coimbra, 3000-548 Coimbra, Portugal; 4 Centro Hospitalar e Universitário de Coimbra (CHUC), 3000-075 Coimbra, Portugal; 5 Nuffield Laboratory of Ophthalmology, NDCN & NIHR Oxford Biomedical Research Centre, University of Oxford, Oxford, OX3 9DU United Kingdom; 6 Laboratory of Cytogenetics and Genomics, Faculty of Medicine, University of Coimbra, 3000-548 Coimbra, Portugal; 7 Faculty of Pharmacy (FFUC), University of Coimbra, 3000-548 Coimbra, Portugal; 8 Centre for Biotechnology, Brock University, St. Catharines, Ontario, L2S 3A1 Canada; 9 Pharmacology and Experimental Therapeutics - Institute of Biomedical Research in Light and Image (IBILI), Faculty of Medicine, University of Coimbra, 3000-354 Coimbra, Portugal; 10 Institute for Nuclear Sciences Applied to Health (ICNAS), University of Coimbra, 3000-354 Coimbra, Portugal; 11 Department of Life Sciences, Faculty of Science and Technology (FCTUC), University of Coimbra, 3000-456 Coimbra, Portugal

## Abstract

Cancer stem cells (CSCs) are a small population of resistant cells inhabiting the tumors. Although comprising only nearly 3% of the tumor mass, these cells were demonstrated to orchestrate tumorigenesis and differentiation, underlie tumors’ heterogeneity and mediate therapy resistance and tumor relapse. Here we show that CSCs may be formed by dedifferentiation of terminally differentiated tumor cells under stress conditions. Using a elegant co-culture cellular system, we were able to prove that nutrients and oxygen deprivation activated non-malignant stromal fibroblasts, which in turn established with tumor cells a paracrine loop mediated by Interleukine-6 (IL-6), Activin-A and Granulocyte colony-stimulating factor (G-CSF), that drove subsequent tumor formation and cellular dedifferentiation. However, by scavenging these cytokines from the media and/or blocking exosomes’ mediated communication it was possible to abrogate dedifferentiation thus turning these mechanisms into potential therapeutic targets against cancer progression.

## Introduction

Tumors are dynamic and heterogeneous entities that act like organs in a perfect trading with the entire body. They are comprised of distinct cell populations that can either be the direct product of cells with different cellular or embryonic origins, or a byproduct of the asymmetric division of stem-like cells. In agreement, cancer-committed stem-like cells, often named CSCs, have been identified virtually in all solid and hematological tumors^1^.

CSCs share several similarities with normal adult stem cells (SCs), including self-renewal capacity, expression of pluripotency surface markers and multilineage differentiation properties^2^, but unlike them, CSCs have sustained cellular proliferation^3^. Their tremendously variable frequency among the different tumor types, and within tumors of the same origin, makes them difficult to ascertain^4^. They were initially thought to develop from the pre-existing normal tissue SCs following exposure to molecules secreted by the tumor^5^, but there is now consensus that CSCs may arise either directly following transformation of normal tissue SCs or by dedifferentiation of non-SCs^6^, for instance following epithelial to mesenchymal transition (EMT)^7,8^, or radiochemotherapy, as recently reviewed by Chen and collaborators^9^.

Exploiting the recently evoked involvement of microenvironment and cytokines and soluble molecules in keeping and inducing CSCs’ phenotype may constitute a new molecule-focused therapeutic opportunity. In this line, using an elegant cell culture model previously developed in the laboratory we were able to show that IL-6, G-CSF and Activin-A released by stromal fibroblasts drive lung carcinoma cells’ dedifferentiation and CSCs formation. Moreover, it was possible to ascertain a specific role to each cytokine as well as to establish the dynamics of the cytokines’ release. The attained results sustain the active role of microenvironment in tumor progression and present a new avenue for therapeutic intervention aiming CSCs ablation and metastasis abrogation.

## Results and Discussion

### *In vivo* cellular derivation increased cells’ malignant potential

**Figure 1:**
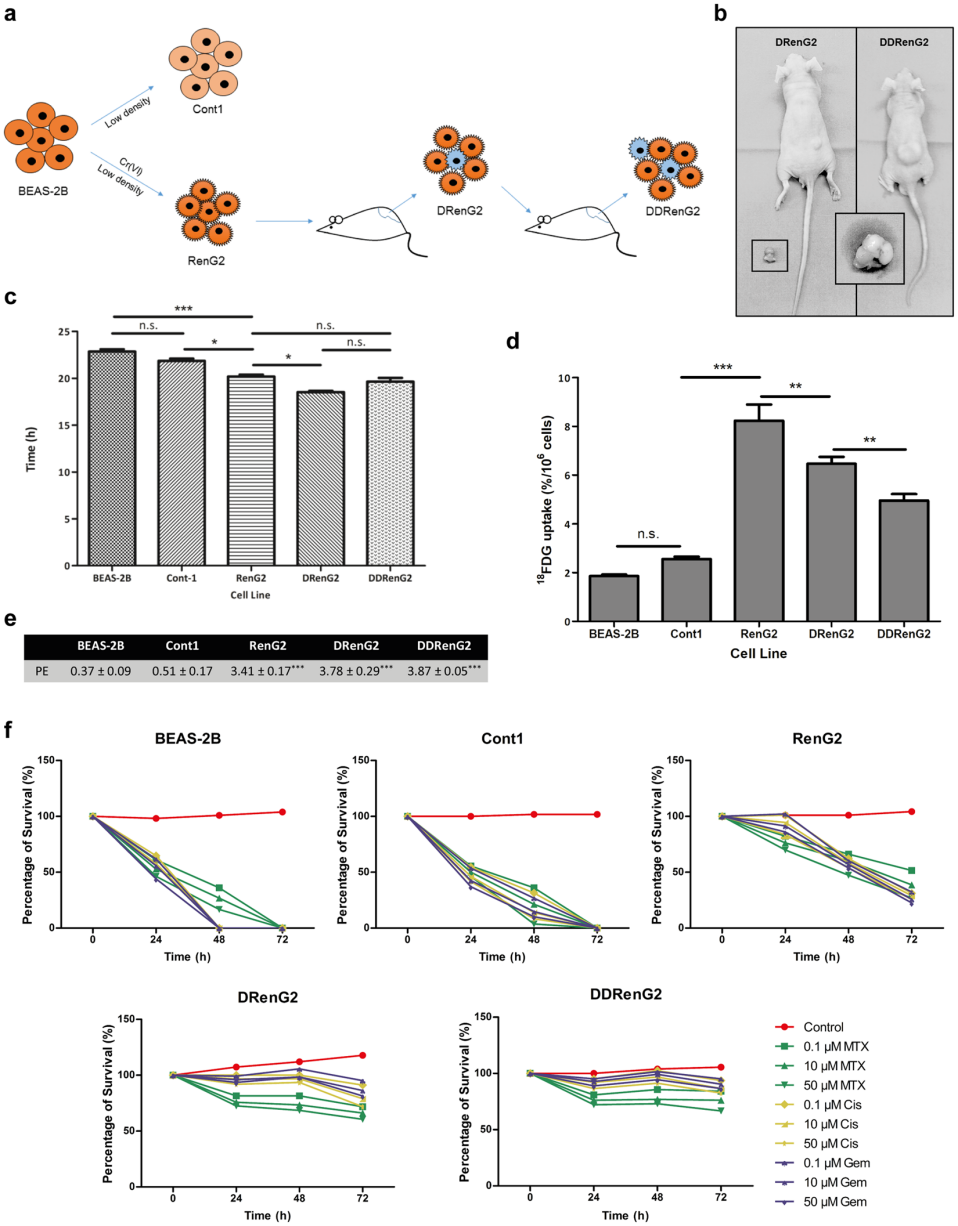
RenG2 cells’ *in vivo* derivation increased their malignant potential. (**a**) Derivation experimental protocol. (**b**) Comparative tumorigenic potential of the derivative cellular systems. Tumors induced by the same number of cells in the same experimental period, clearly depicting DDRenG2′ higher malignant potential. (**c**) Cellular duplication times. Malignant cells replicated significantly faster than their non-malignant progenitors. RenG2 DT was significantly different from that of DRenG2 cells, while no significance was observed when comparing DDRenG2 to its malignant counterparts. (**d**) ^18^FDG uptake. Malignant cells showed a considerably higher glucose uptake. Unexpectedly, however, as malignancy increased the glucose uptake decreased. (**e**) Plating efficiency. Malignant cells exhibited a considerably higher plating efficiency. (**f**) Drug-resistance assays. The higher the degree of malignancy, the higher the resistance to the different drugs, at all tested concentrations Derivative cell lines, in particular, were shown to be more sensitive to MTX than their non-malignant progenitor cells. MTX, methotrexate; Cis, cisplatin; Gem, Gemcitabine. Data represents mean ± SEM. Differences between the means were evaluated either by one-way or repeated measures ANOVA followed by a Bonferroni post test. n.s., no significant; ^*^P ≤ 0.05; ^**^P ≤ 0.01; ^***^P ≤ 0.001. For PE a Bonferroni post test was used.

The malignant RenG2 cell line was established by culturing the non-malignant immortalized human bronchial epithelial cells BEAS-2B at extremely low density in the presence of 1.0 μM hexavalent chromium [Cr(VI)]. This chemical agent was classified by both the IACR and the United States Environmental Protection Agency (USEPA) as a human lung carcinogen of Group I and Group A, respectively^10^, and its concentration was selected based on epidemiologic studies^11,12^ and the observation that it was only slightly cytotoxic^13^. As a control experiment, Cont1 cell line was attained from low-density Cr(VI)-free cultures^14^. Although malignant, RenG2 cells needed about 2 months to induce tumor formation in immunocompromised mice, so their malignant potential was increased by *in vivo* derivation using serial rounds of injection in immunocompromised mice. As a consequence, DRenG2 cells were attained from primary cultures of the RenG2-induced tumor and the DDRenG2 cells from primary cultures of the DRenG2-induced tumor (Fig. 1a). Relative tumorigenic ability comparison confirmed the progressively increased malignancy of the derived systems (Fig. 1b).

Supporting the *in vivo* studies, duplication times’ (DT) calculation showed that the malignant cell lines replicated faster than non-malignant ones, particularly DRenG2 cells which showed a DT of roughly 18.5 h (Fig. 1c). No statistically significant differences were observed between DDRenG2 and either RenG2 or DRenG2 cells, and the results attained for BEAS-2B cells corroborated prior studies of Costa and colleagues by documenting a DT of approximately 23 h^13^. Cont1 cells showed no statistically significant differences in their DTs when compared to BEAS-2B, thus presenting them as a good experimental control.

Consistent with previous observations showing that malignization is accompanied by an increase in glucose uptake and a stimulation of aerobic glycolysis^15–17^, the comparative study of [^18^F]-fluoro-2-deoxyglucose (^18^FDG)-uptake showed that the malignant cell lines had a considerably higher glucose demand than the non-malignant ones (Fig. 1d). However, as malignancy increased the glucose uptake decreased, either illustrating a cellular strategy to ensure surviving, for instance through the overexpression of drug efflux pumps as previously observed^18,19^, or simply indicting the presence of a progressively bigger slow-dividing stem-like cellular population in those cell lines. Moreover, malignant systems displayed a progressively higher clonogenic capacity (Fig. 1e), higher migration ability (Fig. S1) and an increased cell survival following treatment with conventional lung carcinoma-directed drugs, namely cisplatin, methotrexate and gemcitabine (Fig. 1f). In fact, both derivative systems distinctively succeed in surviving the entire repertoire of employed drugs, particularly DDRenG2. Altogether the attained results confirmed the malignant nature of both RenG2 and their progeny by identifying features consensually ascertained to malignant cells.

### Malignant potentiation was underlined by CSCs formation

Medema’s laboratory proposed that only CSCs are endowed with tumorigenic capacity and the ability to resist chemotherapy^20^. By interpreting the previous results in light of Medema’s theory, it became plausible to hypothesize that CSCs mediated BEAS-2B cells’ malignization and were liable for the malignant features of RenG2, DRenG2 and DDRenG2 cell lines. To test this hypothesis the sphere-forming assay (SFA) along with immunocytochemistry was performed. SFA constitutes a reliable method to specifically isolate CSCs from inside a heterogeneous mixture of cells while preserving the key characteristics of the original patient tumors^4^. Immunocytochemistry, instead, allows monitoring EMT, a proposed source of CSCs^21^, as the loss of epithelial features towards a mesenchymal phenotype triggers the expression of α-smooth muscle actin (α-SMA) and increases that of Vimentin^22^.

**Figure 2:**
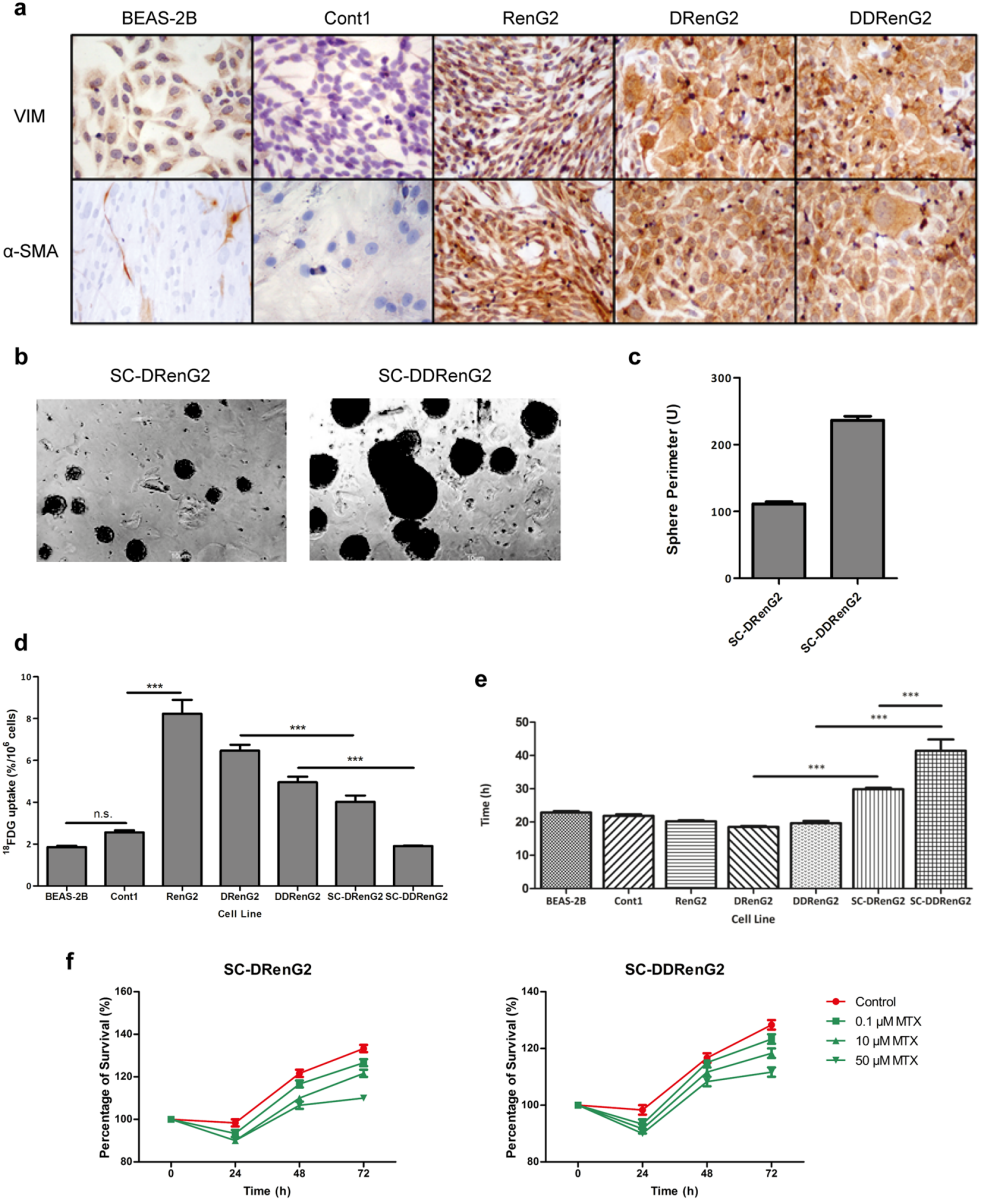
RenG2 cells’ derivation featured CSCs formation by dedifferentiation. (**a**) Immunocytochemistry study of Vimentin and α-SMA. Both BEAS-2B and Cont1 non-malignant systems displayed a basal staining for Vimentin. Conversely, α-SMA staining was negative in these cell lines. All the malignant systems, however, presented a strong staining for both Vimentin and α-SMA. A magnification of 400x was used in all panels. VIM, Vimentin. (**b**) Sphere-forming assay from the derivative systems. DDRenG2 cell line formed more and larger spheres than its progenitor DRenG2 cell line. A magnification of 100x was used in both photographs. (**c**) Perimeter analysis of the spheres formed by both derivative systems. 50 spheres were measured per analyzed cell line. (**d**) Comparative analysis of ^18^FDG uptake. Both SC-DRenG2 and SC-DDRenG2 cell lines uptake significantly less ^18^FDG in comparison to the other malignant cell lines. (**e**) Comparative study of cellular duplication times. Both SC-DRenG2 and SC-DDRenG2 had considerably higher DTs than their derivative progenitors. Moreover, SC-DDRenG2 needed even more time to replicate than SC-DRenG2. (**f**) CSCs’ survival following MTX treatment. MTX not only failed at eradicating CSCs, but also was unable to block their division, as both SC-DRenG2 and SC-DDRenG2 grew in the presence of the drug. Bars represent means ± SEM. Differences between the cell lines’ means were evaluated either by one-way or repeated measures ANOVA followed by a Bonferroni post test. n.s., no significant; ^*^P ≤ 0.05; ^**^P ≤ 0.01; ^***^P ≤ 0.001.

Basal levels of Vimentin staining were found in BEAS-2B and Cont1, illustrating the ubiquitousness of this protein (Fig. 2a). The malignant systems, however, showed an increased expression of Vimentin, thus revealing their mesenchymal phenotype and explaining their increased motility, which according to Mendez and collaborators is the result of the assembly of Vimentin intermediate filaments^23^. Not surprisingly, α-SMA was only expressed in the malignant cell lines, not only corroborating the epithelial nature of both BEAS-2B and Cont1, but also suggesting the stem potential of the malignant systems (Fig. 2a). SFA, however, only yielded spheres when either DRenG2 or DDRenG2 cell lines were cultured at restraining conditions (Fig. 2b,c), and the spheres attained with DDRenG2 cells were not only bigger but also more numerous than those formed by DRenG2 (Fig. 2c). This observation imprinted a higher stem potential to the DDRenG2 cellular system and further suggested that the CSCs isolated from DRenG2 cultures were obtained through dedifferentiation of RenG2 cells and not by transformation of endogenous stem-like cells. The resulting DRenG2 and DDRenG2 spheres were purified after 3 generations of isolation and CSC lines were established out of each of the derivative systems and respectively named SC-DRenG2 and SC-DDRenG2.

New relative characterization of the attained CSCs lines showed that the glucose requirements of the CSC systems were comparable to those of non-malignant BEAS-2B and Cont1 cells, and thus significantly lower than any of the malignant progenitor cell lines (RenG2 cells included) (Fig. 2d). However, MTT-based cell DTs’ calculations revealed that the modest glucose necessities of CSCs portrayed their quiescent status, as these cell populations have a considerably longer cell cycle than their progenitors (Fig. 2e). Furthermore, methotrexate-resistance studies showed that, contrarily to what was observed to the progenitor malignant systems, drug treatment failed to abrogate CSCs’ cycle progression, as the cells kept dividing in the presence of the drug (Fig. 2f). This higher resistance of CSCs to therapy is in line with previous observations^18,24–26^ and is thought to be the main responsible for quiescence^27,28^. In fact, as many chemotherapy agents require cell cycle progression to act, CSCs’ avoid death by entering quiescence and inducing a very efficient activation of the DNA repair genes^6,18,19^.

### Dedifferentiation as a source of CSCs

The confirmation of SC-DRenG2 and SC-DDRenG2 stem potential and the observation that there was a progressive increment in CSCs sub-populations along the derivative systems concomitantly suggested that the mouse subcutaneous compartment microenvironment drove and supported RenG2 cells’ dedifferentiation. To further prove this hypothesis mouse cells were surgically isolated from the thoracoabdominal aponeurosis of the animals and Transwell® (CLS3450, Corning®) co-cultured with RenG2 cells for 8 weeks (the same period RenG2 cells needed to induce tumor formation in immunocompromised mice). After co-culture, CSCs were searched for and positively isolated from the RenG2 population using the SFA, and named iRenG2. The formation of spheres was observed soon after cells’ platting, similarly to what was previously seen for both SC-DRenG2 and SC-DDRenG2.

**Figure 3:**
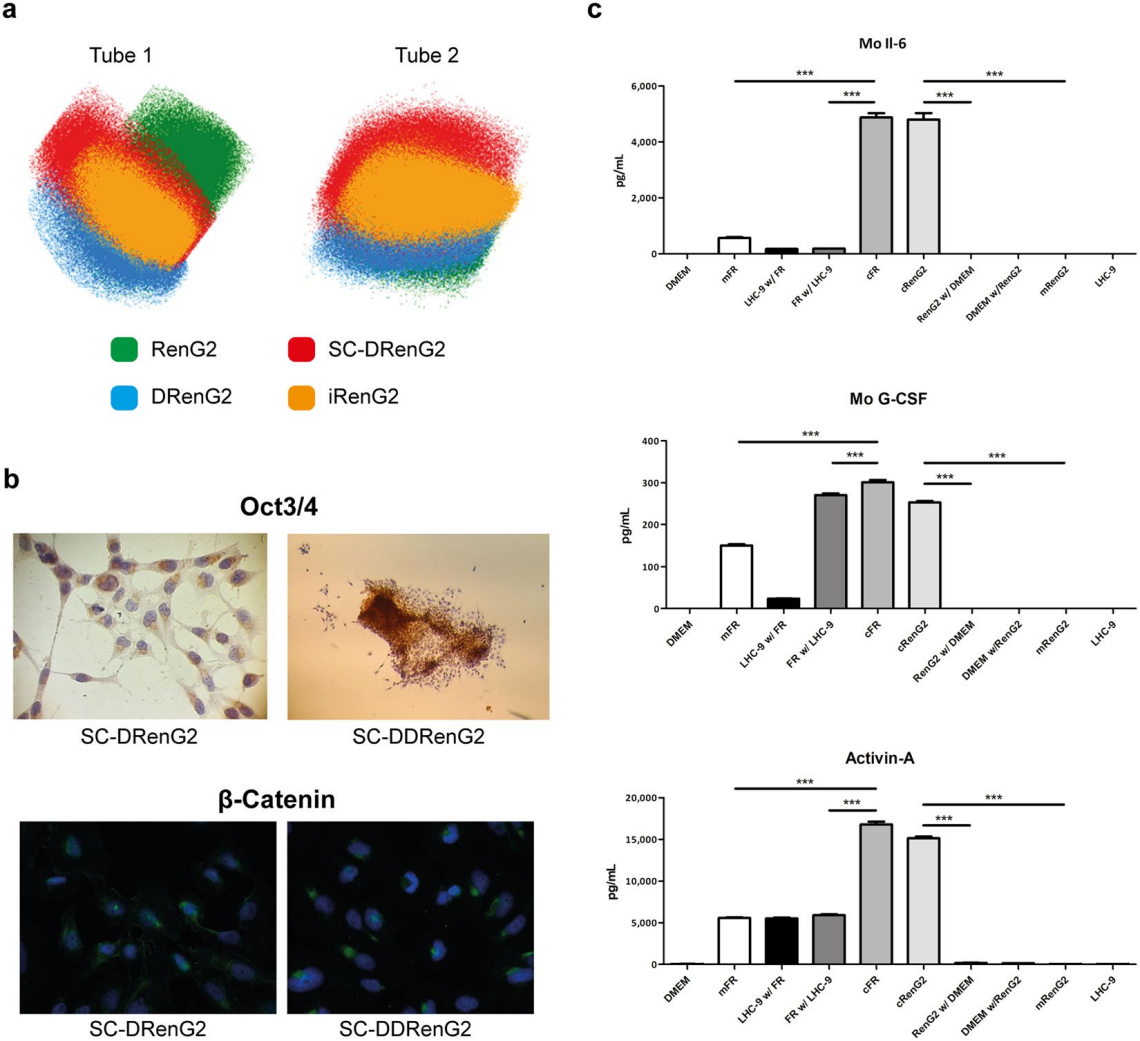
Isolated CSCs’ depicted classical stem properties and were attained by a cytokine-mediated paracrine loop established between the tumor cells and the microenvironment. (**a**) Flow cytometry scattering plots comparing the iRenG2 cell line to RenG2, DRenG2 and SC-DRenG2. In both tubes the yellow-represented iRenG2 cells were more close to both DRenG2 and SC-DRenG2 than to RenG2, illustrating their closer identity. Colored dots represent individual cells. RenG2 green, DRenG2 light blue, SC-DRenG2 red and iRenG2 yellow. (**b**) Immunocytochemistry study of Oct 3/4 and β-Catenin. Both CSCs systems depicted a marked staining of both proteins, with β-Catenin preferentially localized to the nuclei. A magnification of 400x was used in all panels. (**c**) IL-6, G-CSF and Activin-A levels in the conditioned media of the RenG2-FR co-culture. The cytokines’ levels were significantly increased in the co-cultures relative to the controls. The use of an anti-mouse antibody allowed the detection of FR-produced cytokines in the upper compartment. Mo, mouse; mFR, monocultured FR cells; cFR, FR cells co-cultured with RenG2 cells; cRenG2, RenG2 cells co-cultured with FR cells; mRenG2, monocultured RenG2 cells; w/, co-cultured with; n.s., no significant. Data represent means ± SEM. Differences between the means were evaluated by one-way ANOVA followed by a Bonferroni post test.

To compare the isolated iRenG2 cells with their progenitors RenG2 cells and both DRenG2 and SC-DRenG2, panels of malignancy-associated genes (Fig. 3a) and molecular markers (Fig. 3b) were selected. The attained results showed that iRenG2 cells’ molecular signature, unlike their progenitor RenG2, was more similar to that of both DRenG2 and SC-DRenG2, thus confirming microenvironment-mediated dedifferentiation of the RenG2 cells and establishing the process as paracrine mediated in nature. Final confirmation was attained from cytokine multiplex array (BioRad®) and ELISA performed in the conditioned media of the co-cultures, which identified consistently increased levels of IL-6, G-CSF and Activin-A (Fig. 3c). A proof of concept experiment was also performed to confirm the action of these cytokines over RenG2 cells by mono-culturing these cells in their presence, and the acquisition of stem properties was positively documented (Fig. S2). Moreover, the attained results were reproduced using human bronchial fibroblasts (HBFs) attained out of a fresh non-malignant human lung sample, and the same transformation was observed.

### Dedifferentiation-implicated cytokines were transported inside exosomes

Paracrine communication is by definition the activation of cellular signaling pathways mediated by soluble factors, which may either be freely released to the media or transported as cargos of extracellular vesicles. Among these vesicles, exosomes in particular are microvesicles of unique characteristics and composition that encapsulate material from cells’ cytoplasm, thus protecting it against harsh extracellular environments^29,30^.

**Figure 4:**
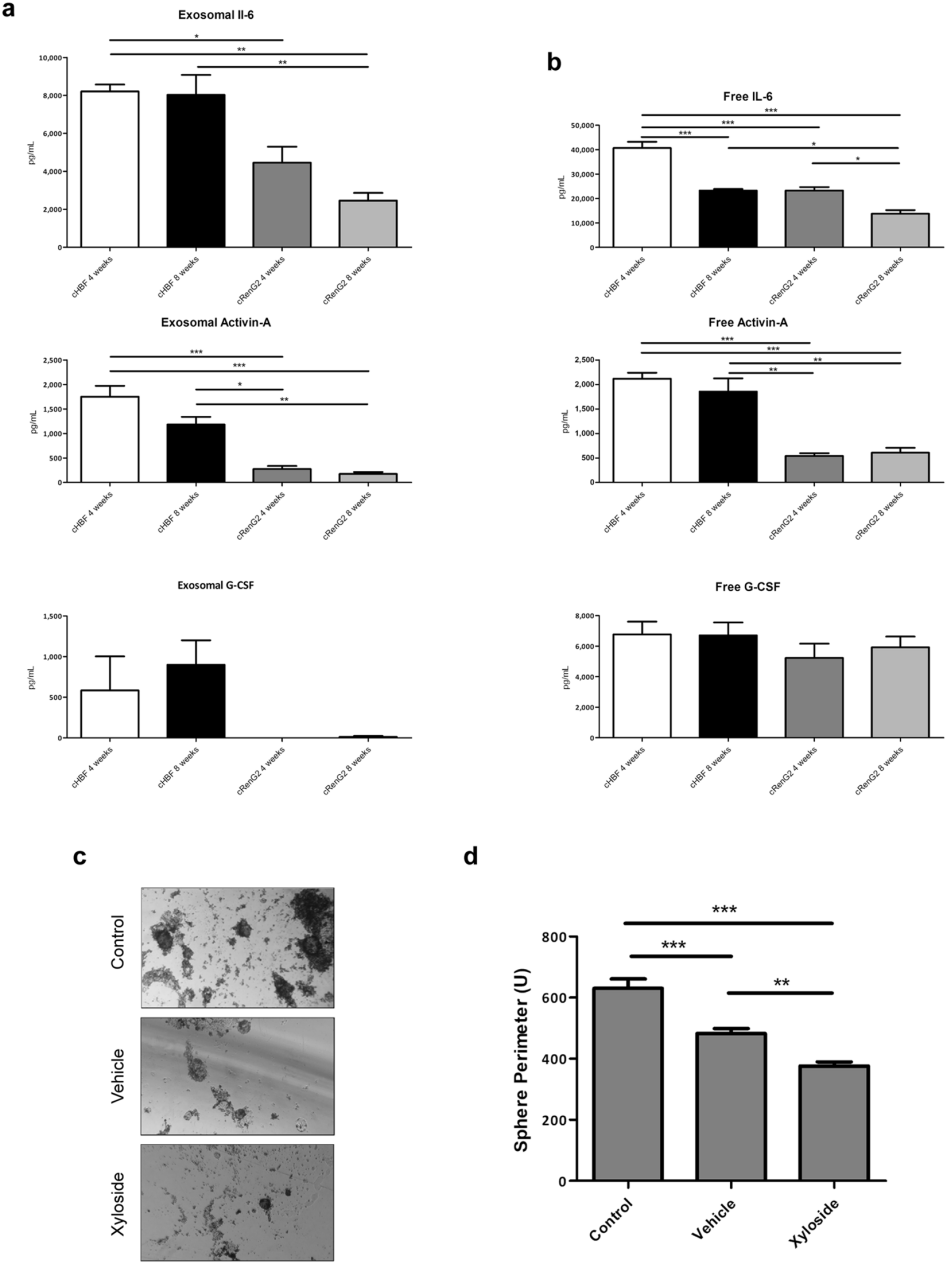
Exosomes mediated the communication between tumor and stroma cells. (**a**) Exosomes’ content analysis. IL-6 and Activin-A-containing exosomes were present in the upper compartment. (**b**) Cytokine levels in the co-cultures’ conditioned media. Cytokines’ levels were maintained relatively stable along time in co-culture. (**c**) SFA of RenG2 cells after co-culture with HBF in the presence of xyloside. A magnification of 100x was used in all panels. (**d**) Perimeter analysis of the attained spheres. 10 spheres were measured per analyzed cell line. There was a significant reduction in the sphere-formation ability, resulting from the xyloside-mediated abrogation of exosome communication. Data represent means ± SEM. Differences between the means were evaluated by one-way ANOVA followed by a Bonferroni post test.

Hypothesizing that exosomes were involved in RenG2 cells’ dedifferentiation, these microvesicles were isolated from the conditioned media of the long-term co-cultures of HBFs and RenG2 cells, and their content screened. The attained results showed the presence of exosomes containing all three cytokines in both compartments, with higher exosome levels in the bottom compartment, thus demonstrating that cytokine-containing exosomes were being secreted by the fibroblasts, and that these microvesicles were able to trespass the membrane of the inserts. An exception should be made for G-CSF whose exosomes were not found in the upper compartment possibly because this cytokine is produced in smaller amounts and/or because it is only needed in the latter steps of the dedifferentiation process, as afterwards proposed. The levels of IL-6, on the other hand, were significantly higher than those of the other cytokines in any of the compartments (Fig. 4a). The presence of the cytokines as free molecules in the conditioned media was also assessed to evaluate the impact of an eventually non-exosome mediated release. Results demonstrated higher concentrations of all three cytokines in both compartments when compared to exosome levels, which yet remained relatively stable along time (Fig. 4b). Performing the abovementioned co-culture experiments in the presence of the exosome-uptake blocker xyloside yielded a definitive proof of the exosome-mediated cytokines’ transport and of their action over RenG2 cells. Xyloside is a small hydrophobic compound that inhibits proteoglycans’ biosynthesis and whose use as an exosome-uptake blocker is quite recent^31^. Its presence in the current co-culture system abrogated the acquisition of CSCs traits by RenG2 cells, which resulted in a dramatically reduction of these cells’ ability to form spheres (Fig. 4c,d).

### IL-6 and Activin-A are directly involved in dedifferentiation, while G-CSF is implicated in keeping the stem phenotype

To fully understand the dynamics of the dedifferentiation process, the impact of each individual cytokine in the overall communication process was ascertained. To this end neutralizing antibodies against IL-6, G-CSF and Activin-A were used to scavenge cytokines from the co-cultures’ media, either alone or in combinations.

**Figure 5:**
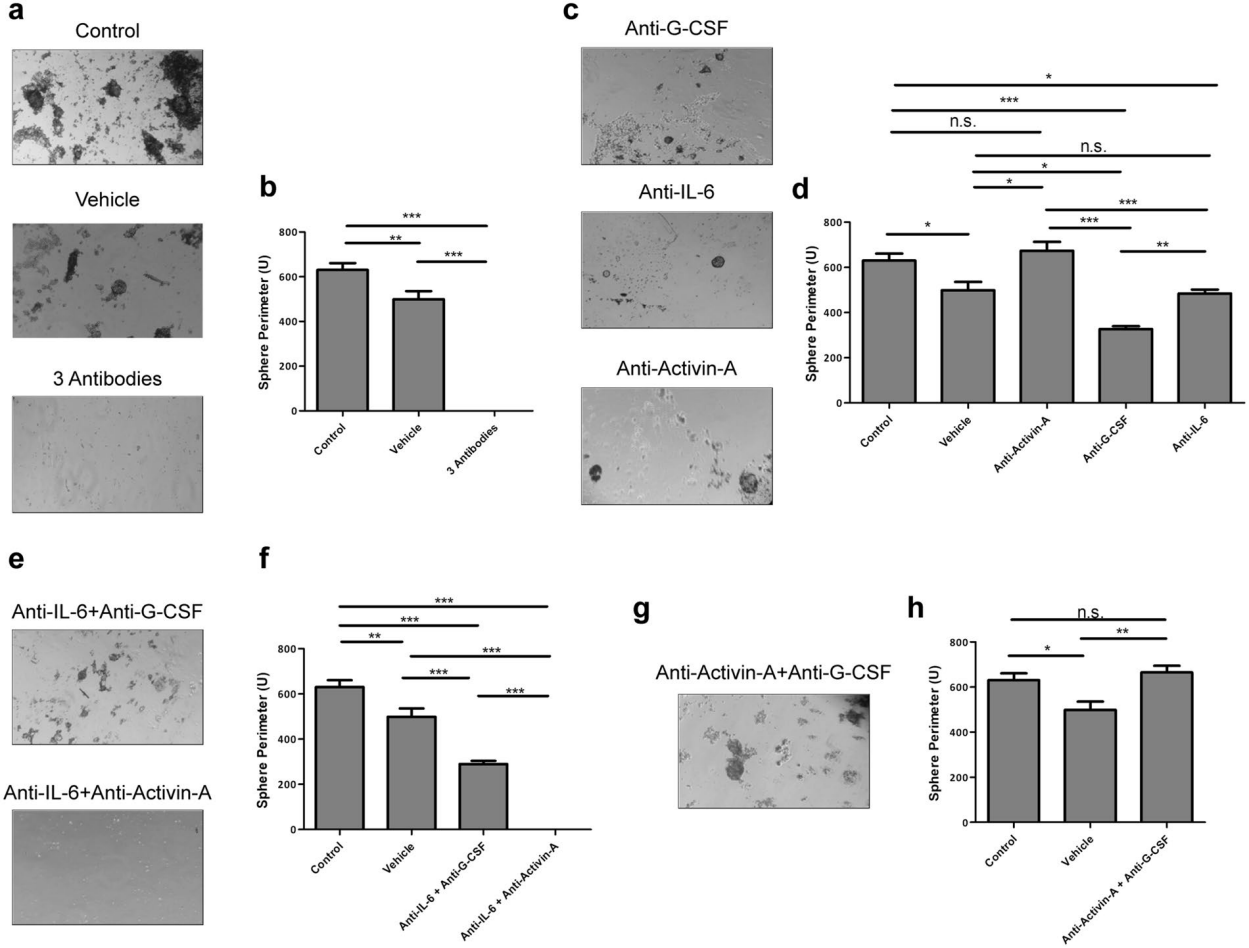
IL-6 and Activin-A are the actual drivers of dedifferentiation. (**a**) SFA of RenG2 cells after co-culture with HBF in the presence of neutralizing antibodies against IL-6, G-CSF and Activin-A. No spheres were formed. (**b**) Perimeter analysis. (**c**) SFA of RenG2 cells after co-culture with HBF in the presence of neutralizing antibodies against IL-6, G-CSF and Activin-A, individually. Spheres were formed in all conditions, being bigger in the condition where both IL-6 and G-CSF were present in the culture media. (**d**) Perimeter analysis. (**e**) SFA of RenG2 cells after co-culture with HBF in the presence of only either Activin-A or G-CSF. Activin-A was able to induce sphere formation, while G-CSF alone was unable to do it. (**f**) Perimeter analysis. Spheres formed by Activin-6 were lesser and smaller than those formed when IL-6 was present. (**g**) SFA of RenG2 cells after co-culture with HBF in the presence of only IL-6. (**h**) Perimeter analysis. IL-6 was the most potent inducer of dedifferentiation. A magnification of 100x was used in all panels. 10 spheres were measured per perimeter analysis for each cell line. Data represent means ± SEM. Differences between the means were evaluated by one-way ANOVA followed by a Bonferroni post test.

Corroborating the previous observations, whenever all the three cytokines were scavenged from the media, sphere formation was abrogated (Fig. 5a,b). Also, the independent scavenge of each of the three cytokines failed to block dedifferentiation, thus showing that at least one of the three is necessary to trigger the process (Fig. 5c,d). However, the concomitant neutralization of Activin-A and IL-6 resulted in no sphere formation, while the simultaneous neutralization of IL-6 and G-CSF yielded smaller and fewer spheres than the control co-cultures (Fig. 5e,f). These observations showed that only IL-6 and Activin-A were endued with the ability to trigger dedifferentiation, and that IL-6 was the more potent inducer of the process. Nonetheless, they also suggested that despite the fact that Activin-A is able to induce CSCs’ formation, it seems that this cytokine might also act as a differentiation inducer of the pre-formed CSCs. In agreement, whenever Activin-A was present, the number of spheres was reduced, exception made to the situation where only this cytokine was present. Corroborating this hypothesis are several reports in the literature indicating Activin-A as a differentiation inducer^32–34^.

IL-6 was the strongest inducer of dedifferentiation as more and bigger spheres formed when this cytokine was solely present (Fig. 5g,h). This observation is in line with many recent studies performed in different tumor types, namely, gastric^35^, breast^36^ and bone^37^, reporting that not only IL-6 is indeed able to induce dedifferentiation, but also that it does so through the activation of STAT3 and consequently, of the Notch signaling pathway^38^. The presence of G-CSF in the co-culture system, although not necessary for the dedifferentiation process, sustained CSCs’ proprieties in previously developed CSCs’ pools (Fig. 5c–f). This result corroborates Agarwal and colleagues’ work that showed that G-CSF sustained neuroblastoma CSCs’ pool through a STAT3 mechanism^39^.

## Final Integration and Conclusion

Microenvironment-coordinated tumor biology has been a very active field of research in the past decade. Many reports in the literature have highlighted its essential role not only on tumor support but also on the early steps of tumorigenesis^40^. More recently microenvironment has been proved crucial in metastatic site definition after an intricate across-body cytokine-mediated crosstalk between the primary tumor and the bone marrow had been implicated in pre-metastatic niche preparation^41^.

**Figure 6:**
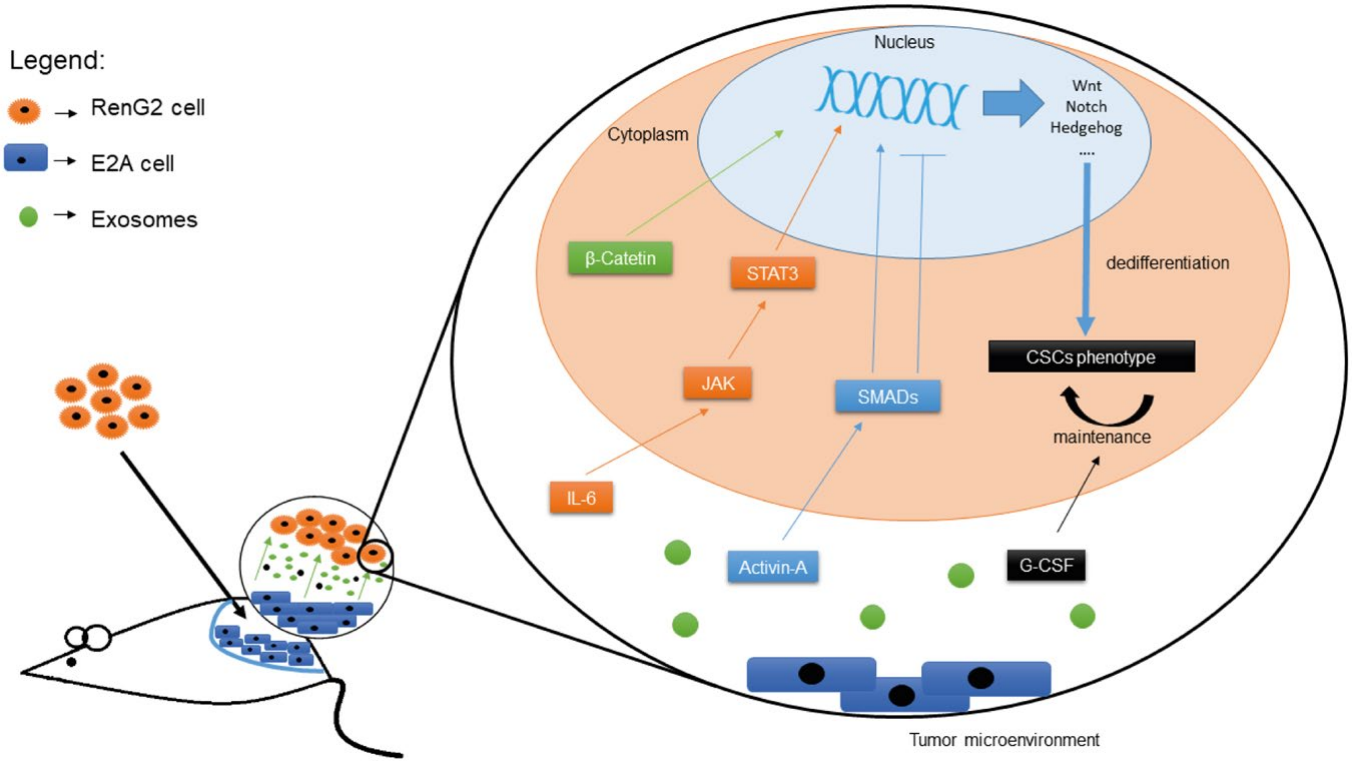
Explanatory model for microenvironment-driven dedifferentiation. Fibroblasts-released exosomes containing IL-6, Activin-A and G-CSF, either combined or separated, interacted with the tumor cells inducing alterations in DNA expression, most probably through STAT3 and Smad activation. The consequent activation of stemness-associated pathways such as Wnt, Notch and Hedgehog drove tumor cells’ dedifferentiation, which was subsequently maintained by the activity of G-CSF. Activin-A seemed to act as a sensor of the CSCs’ pool homeostasis, inducing CSCs’ differentiation whenever a certain threshold was reached.

Aiming to shed some light on the role played by CSCs in the overall tumorigenic process, the present work endorsed a model for CSCs’ dedifferentiation in which the tumor-mediated co-option of non-malignant microenvironment cells leads to an increase in IL-6 and Activin-A levels in the tumor microenvironment, which in turn drive tumor cells’ dedifferentiation, and consequently, CSCs’ formation. Following dedifferentiation, Activin-A maintains CSCs’-pool homeostasis, inducing differentiation whenever it overcomes a certain threshold, and G-CSF provides the CSCs’-niche with the appropriate conditions to sustain the undifferentiated phenotype of its cells, by acting downstream of the previous cytokines (Fig. 6).

The identification of this and other cytokine loops, as for instance the one recently identified in breast cancer and mediated by Granulocyte-macrophage colony-stimulating factor (GM-CSF) and Chemokine (C-C motif) ligand 18 (CCL18)^42^, opened a new branch of cancer therapies targeting the microenvironment. It is expected that in the following years subsequent studies identify molecules to modulate these communication pathways that in combination with the conventional or eventually new therapy protocols, hamper tumor progression.

## Methods

### Cells and Cell Culture

RenG2 cells are a malignant cellular system previously produced in our laboratory^14^, maintained in LHC-9 medium (Gibco®) at the initial cellular density of 0.1 × 10^6^ cells/cm^2^, unless otherwise stated. The derivative (DRenG2 and DDRenG2) and the stem (SC-DRenG2, SC-DDRenG2 and iRenG2) systems were attained as abovementioned and kept in DMEM:F12 (1:1) medium (Gibco®) supplemented with 5% penicillin (5000 U/mL)-streptomycin (5000 μg/mL) (Gibco®), 5% Insulin-Transferrin-Selenium pyruvate (ITS) solution (Gibco®), 0.1% amphotericin (Gibco®), 0.6 g of sodium bicarbonate (Sigma-Aldrich®) and 0.08 g of putrescine (Sigma-Aldrich®).

FR primary cell line was attained using a protocol adapted in our laboratory by surgically removing cells from the thoracolumbar aponeurosis of BALB/c-nu/nu mice (Charles-River). Isolated tissue was fragmented, and the attained small pieces distributed throughout the basis of a cell culture flask (SPL-Biosciences®). A drop of fetal bovine serum (FBS) (Gibco®) was added to each of the fragments to help them adhering and to provide them with nutrients. Finally, the flask was turned upside-down and DMEM medium (Gibco®) supplemented with 10% FBS (Gibco®) was added to the top surface of the flask. Fragments were allowed to attach upside-down for 24 h and then the flask was gently turned to the up right. After monolayer formation the cells were disaggregated, sub-cultured and amplified, yielding the FR cellular system. HBF cell line was developed using the same protocol but from non-malignant human lung tissue attained from a patient at the CHUC, through appropriate informed consents and according with the ethical procedures approved by the Ethical Committee of the Faculty of Medicine of the University of Coimbra. Only redundant tissue was submitted to research. Routine procedures were followed, fulfilling the current criteria in Pathology for diagnosis, prognosis and treatment.

For co-culture experiments either FR or HBF cells were cultured in a 6 well plate (SPL-Biosciences®) equipped with 4.5 cm^2^ Transwell® insert (CLS3450, Corning®) containing RenG2 cells.

### Tumorigenic Assays

*In vivo* tumorigenic assays were performed in NOD/SCID IL2Rγnull mice (Charles River) by subcutaneously injecting 5 × 10^6^ cells into the flank region of the animals. Mice were housed under standard conditions at the CNC animal facility and screened twice a week for tumor formation. All animal procedures were conducted according to the EU Directive 2010/63/EU for animal experiments and reviewed and approved by DGAV, ORBEA and the animal facility ethics committee.

### Duplication Times Calculation

Duplication times were attained using previously established MTT assay protocols^43^ and calculations were performed in the Doubling Time software at Doubling Time webpage^44^.

### ^18^FDG uptake

1.5 mL of single-cell suspensions containing 2 × 10^6^ cells/mL were attained from either adherent-growing cell lines or tridimensional spheres. The suspensions were placed in 10 mL centrifuge tubes (SPL-Biosciences®) and left for recovery for 1 h at 37 °C. Subsequently, a calculated volume of 37 °C-heated ^18^FDG was added to reach a final concentration of 0.75 MBq/m and tubes were homogenized and conserved at 37 °C. After 1 h incubation, samples of 200 μL were collected to 1.5 mL microcentrifuge tubes (SPL-Biosciences®) containing 500 μL of ice-cold PBS (Sigma-Aldrich®). Tubes were then centrifuged 1 min at 10 000 rpm and the supernatants were collected into glass tubes. 500 μL of ice-cold PBS (Sigma-Aldrich®) were added to the pellets to wash any remaining radioactive medium and tubes were again centrifuged. Supernatants were collected to the same glass tube as previous and cell pellets were preserved. Finally, both the supernatants and the pellets were assayed for radioactivity using a Radioisotope Calibrator Well Counter (CRC-15W Capintec) narrowed to the ^18^F sensitivity energy window (400–600 keV). All cell populations were studied in triplicate in at least three sets of independent experiments. The attained results represent the percentage of cells’ radioactivity relatively to the total radioactivity added, normalized per million of cells.

### Clonogenic Assays

13 cells/cm^2^ cells were plated onto 100 mm Petri dishes (SPL-Biosciences®), allowed to grow for 15 days and then fixed and stained with crystal violet (Sigma-Aldrich®) according to the protocol established by the group of van Bree (39)^45^. Surviving colonies containing more than 10 cells were scored to assess cloning efficiency and the complete protocol was repeated at least three times. Plating efficiency (PE) was calculated dividing the number of colonies formed by the number of cells seeded and the results were presented as a mean ± SEM of three independent assays.

### Scratch Migration Assay

4 × 10^3^ cells/cm^2^ were added to 60 mm cell culture dishes (SPL-Biosciences®) and allowed to reach confluence. After a linear scratch was performed using a p200 pipet tip, the cultures were washed and an experimental site was defined in each dish. Cells were allowed to grow, and photographs were taken at 0 h, 12 h, 19 h, 24 h, 27 h, 37 h, 49 h, 60 h, 73 h, 82 h, 93 h, 176 h and 200 h using a Moticam 2300 3.0 M Pix- el USB 2.0 camera (Motic) coupled to a AE31 microscope (Motic).

### Drug-resistance Assays

Single-cell suspensions of all the five cell lines proliferating or attained from the dissociations of spherical clones were plated into a 24-well plates at an optimized initial cellular density of 8 × 10^3^ cells/cm^2^. 24 h after cells’ seeding, 10 μL of each drug solution [cisplatin (Cis, CG6413, Generis®), methotrexate (MTX, Teva Pharmaceutical Industries) and gemcitabine (Gem, Gemzar®, Lilly)] were administered to attain the desired final concentration (0.1 μM, 10 μM and 50 μM). For each condition, including the controls, three independent assays were carried in triplicate. Cells’ viability was assessed every 24 h, during 3 days using the MTT reduction assay.

### CSCs’ isolation – Sphere Formation Assay

CSCs’ isolation was performed using the SFA. To that end low adherence 6-well plates (SPL-Biosciences®) were prepared by coating the plates’ surface with a 2% poli-(2-hydroxyethyl methacrylate) (poli-HEMA). The isolation medium consisted of a 1:1 mixture of the CSCs maintaining medium with a 2% methylcellulose (Sigma-Aldrich®) solution. For the isolation, 2 mL of a cellular suspension containing 3 × 10^4^ cells/mL were added to each well and the isolation medium was supplemented with 10 ng/mL of both human EGF (Sigma-Aldrich®) and bFGF (PeproTech®). Cells were allowed to grow and supplements’ concentration was replaced every 2 days. Spheres formation was accompanied and photographed along time, and 15 day after platting they were collected, washed with PBS, and plated in T_25_ cell culture flasks (SPL-Biosciences®) provided with 5 mL fresh maintaining medium. Cells were allowed to attach and expand, and the protocol of isolation was repeated twice when they reached nearly 80% confluence.

### Immunocytochemistry

4 × 10^3^ cells/cm^2^, either attained directly from culture flasks or derived from fresh 3^rd^ generation disaggregated spheres (in the case of SC-DRenG2 and SC-DDRenG2 cells), were seeded on the top of microscope slides (VWR) placed inside a 100 mm cell culture dish (SPL-Biosciences®) and cells were allowed to grow until approximately 80% confluence. Following medium aspiration the slides were rinsed twice with PBS (Sigma-Aldrich®), collected into centrifuge tubes (SPL-Biosciences®) containing 50 mL of 95% ethanol (Sigma-Aldrich®) and kept overnight at 4 °C. To quench the endogenous peroxidase activity 15 min incubation was performed in a 3% hydrogen peroxide (H_2_O_2_) solution. Subsequent preparation steps were performed using the Ultra Vision Kit (Thermo Scientific®) according to manufacturers’ instructions. After dehydration, slides were mounted using the Tissue-Tek Glas Mounting Medium (1408, Sakura).

Vimentin was stained using the Vim3B4 primary antibody (Dako Corporation), α-smooth muscle actin the αSM-1 (Leica Biosystems), OCT3/4 with the N1NK (Leica Biosystems) and β-catenin with CAT-5H10 (ThermoFisher Scientific). DAPI staining was used to identify the nuclei. Cells’ were observed in a Nikon Eclipse 80i microscope and photographs were taken using a Nikon Digital DXM1200F coupled camera.

### Flow Cytometry-based Cellular Characterization

Four different cytometry tubes containing 300 μL of 1 × 10^5^ cells single-cell suspensions were prepared per cellular system, two tubes for the blank controls and the others to be incubated with the selected panel of fluorescence-labeled monoclonal antibodies (mABs) as schematized in Table 1. The mABs used were CD31 (WM59, BD Biosciences), NGFR (C40-1457, BD Biosciences), CD14 (M5E2, BD Biosciences), CD13 (Immu103.44, Beckman Coulter), CD133 (293C3, Miltenyi Biotec), CD11b (ICRF44, BD Biosciences), CD45 (HI30, Invitrogen), CD106 (51-10C9, BD Biosciences), CD105 (1G2, Beckman Coulter) and HLA A, B, C (G46-2.6, BD Biosciences). The volumes of each mAB were selected according to manufacturer’s recommendations and are listed in Table 1.

**Table 1:** Markers and fluorophore used in the flow cytometry-based cellular characterization studies.

	Tube 1		Tube 2	
FITC	CD31	10 µL	CD106	10 µL
PE	NGFR	10 µL	CD30	10 µL
PerCP5.5	CD14	2.5 µL	—	—
PeCy7	CD13	2.5 µL	CD13	2.5 µL
APC	CD133	10 µL	HLA-A,B,C	10 µL
PB	CD11b	2.5 µL	CD11	2.5 µL
PO	CD45	2.5 µL	CD45	2.5 µL

FITC - Fluorescein isothiocyanate; PE - Phycoerythrin; PerCP - Peridinin-cholophyll-protein complex; PeCy7 - Phycoerythrin Cy7-conjugated; APC - Allophycocyanin; PB - Pacific blue; PO - Pacific orange.

Cells were incubated 15 min with the mABs in the dark at RT, rinsed with 2 mL of PBS (Sigma-Aldrich®) and centrifuged for 5 min at 1500 rpm. Pellets were ressuspended in circa 200 μL of the supernatant and sample readings were carried out in a FACS Canto II Flow Cytometer (BD Biosciences). The attained results were analyzed using the CellQuest software (BD Biosciences).

### Enzyme-linked Immunosorbent Assay (ELISA)

ELISAs were performed following manufacturers’ instructions using the Human/Mouse/Rat Activin-A Quantikine ELISA Kit (#DAC00B, R&D Systems), the Human IL-6 Quantikine ELISA Kit (#D6050, R&D Systems) and the Human G-CSF Quantikine ELISA Kit (#DCS50, R&D Systems).

### Multiplex Analysis

FR cells-derived cytokines were searched for performed in the conditioned media of the co-cultured cells using the Bio-Plex ProTM Mouse Cytokine 23-plex Assay Kit (#M60-009RDPD, BioRad), according to manufacturers’ instructions. Samples were studied in triplicate in a Bio-Plex® 200 System (BioRad), and the attained results were analyzed using the Bio-Plex ManagerTM Software, Standard Edition (BioRad).

### Exosomes’ Isolation, Permeabilization and Uptake Blockage

Exosomes’ isolation was performed using the protocol established by Raposo and collaborators^46^. Briefly, successive centrifugations at increasing speed were used to eliminate large cellular debris and the final supernatant was ultra centrifuged at 100 000 G for 70 min to pellet the exosomes. The pellet was then washed abundantly with PBS (Sigma-Aldrich®) to eliminate contaminating proteins, and centrifuged one last time at the same high speed^47^.

Permeabilization followed the protocol designed by Subra and colleagues^48^, according to which 50 μg of exosomal protein were incubated with 5 μL protease inhibitor cocktail (Sigma-Aldrich®) in 1 mL of PBS (Sigma-Aldrich®) for 10 min at RT, and then sonicated 2 × 10 seconds (VWR Ultrasonic Cleaner).

Exosomes’ uptake blockage was attained by adding a 50 mg/mL xyloside alcoholic solution to the cell culture medium. The solution was attained by dissolving 0.1 g of xyloside (Sigma-Aldrich®) in 2 mL of methanol (Sigma-Aldrich®).

### Cytokines Antibody-mediated Blockage

The manufacturer-provided neutralization range and the previously accessed amount of cytokines present in the co-culture media allowed the establishment of the antibodies’ final concentrations. Concretely, 0.6, 0.144 and 0.272 µg/mL of anti-IL-6, anti-Activin-A, anti-G-CSF, respectively, were used. Co-cultures were established using the antibodies alone or in combinations of two or three antibodies. Control co-cultures were also established using the antibodies’ vehicle, 1x PBS.

### Statistical Analysis

Unless stated otherwise, results derive from at least three independent experiments carried out in triplicate, and their statistical analysis was carried out using the Graph Pad Prism software version 7 (GraphPad Inc.). Error bars indicate ± SEM between biological replicates. Statistical significance of multiple-group comparisons was attained using either one-way or repeated measures ANOVA with Bonferroni post hoc analysis. A *p* value < 0.05 was defined as the threshold of significance and the *P* value was categorized according to their interval of confidence.

### Data Availability

No datasets were generated or analyzed during the current study.

## Electronic supplementary material

Supplementary Information
