# Supplementary material for: Stroma-derived IL-6, G-CSF and Activin-A mediated dedifferentiation of lung carcinoma cells into cancer stem cells
Source: Sci Rep. 2018 Aug 1;8:11573. doi: 10.1038/s41598-018-29947-w (PMC6070555; doi:10.1038/s41598-018-29947-w)
Supplement: Supplementary file 1 — Supplementary Information [file 41598_2018_29947_MOESM1_ESM.pdf]

Research Article

Title: Stroma-derived IL-6, G-CSF and Activin-A mediated dedifferentiation of lung carcinoma cells into cancer stem cells

Carlos F.D. Rodrigues[a,b,c,d], Eurico Serrano[a,b], Maria I. Patrício[e], Mariana M. Val[b,f], Patrícia Albuquerque[a,g], João Fonseca[h], Célia M.F. Gomes[b,i], Antero J. Abrunhosa[j], Artur Paiva[d], Lina Carvalho[b,c,d]; M. Filomena Botelho[b,c], Luís Almeida[a,g], Isabel M. Carreira[a,b,c,f] and Maria Carmen Alpoim[a,b,k,*]

[a] Center for Neuroscience and Cell Biology (CNC), University of Coimbra, 3004-517 Coimbra, Portugal;
[b] Center of Investigation in Environment, Genetics and Oncobiology (CIMAGO), Faculty of Medicine, University of Coimbra, 3001-301 Coimbra, Portugal;
[c] Faculty of Medicine (FMUC), University of Coimbra, 3000-548 Coimbra, Portugal;
[d] Centro Hospitalar e Universitário de Coimbra (CHUC), 3000-075 Coimbra, Portugal;
[e] Nuffield Laboratory of Ophthalmology, NDCN & NIHR Oxford Biomedical Research Centre, University of Oxford, Oxford OX3 9DU, United Kingdom;
[f] Laboratory of Cytogenetics and Genomics, Faculty of Medicine, University of Coimbra, 3000-548 Coimbra, Portugal;
[g] Faculty of Pharmacy (FFUC), University of Coimbra, 3000-548 Coimbra, Portugal;
[h] Centre for Biotechnology, Brock University, St. Catharines, Ontario, Canada L2S 3A1;
[i] Pharmacology and Experimental Therapeutics - Institute of Biomedical Research in Light and Image (IBILI), Faculty of Medicine, University of Coimbra, 3000-354 Coimbra, Portugal;
[j] Institute for Nuclear Sciences Applied to Health (ICNAS), University of Coimbra, 3000-354 Coimbra, Portugal;
[k] Department of Life Sciences, Faculty of Science and Technology (FCTUC), University of Coimbra, 3000-456 Coimbra, Portugal;

***Corresponding Author:**
Maria Carmen Alpoim
Departamento de Ciências da Vida
Faculdade de Ciências e Tecnologia
Universidade de Coimbra
Calçada Martim de Freitas
3000-456 Coimbra
Portugal
Phone: +351239240726
Fax: +351239 240 701
E-mail: mcalpoim@bioq.uc.pt

Supplementary Information

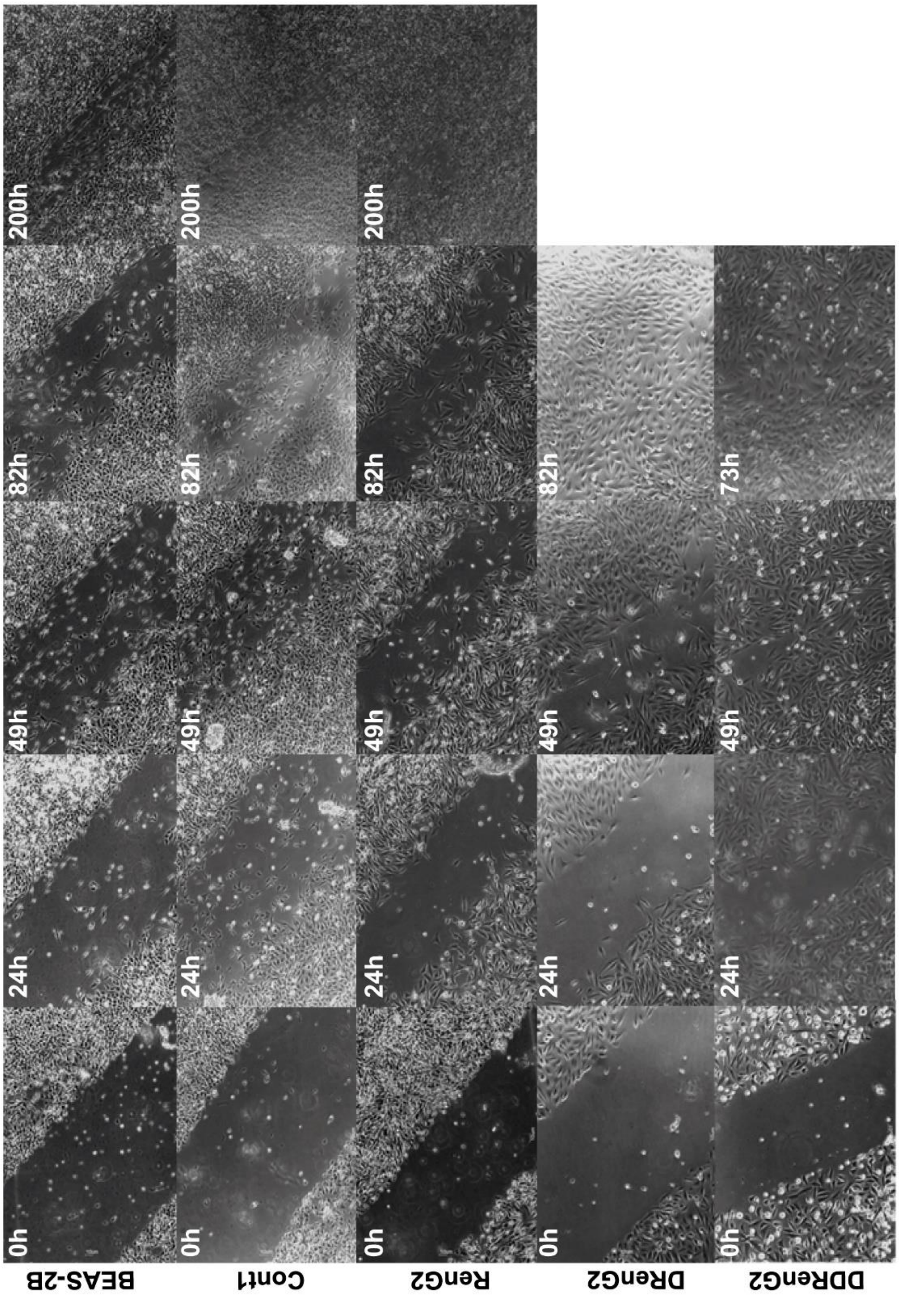

Supplementary Figure S1 – Migration ability of the different cellular systems. Both BEAS-2B and Cont-1 cell lines failed to close the scratch, even after 200 h. RenG2 cells grossly closed it, but DRenG2 and DDRenG2 did it faster and better in progressively less time. A magnification of 100x was used in all panels.

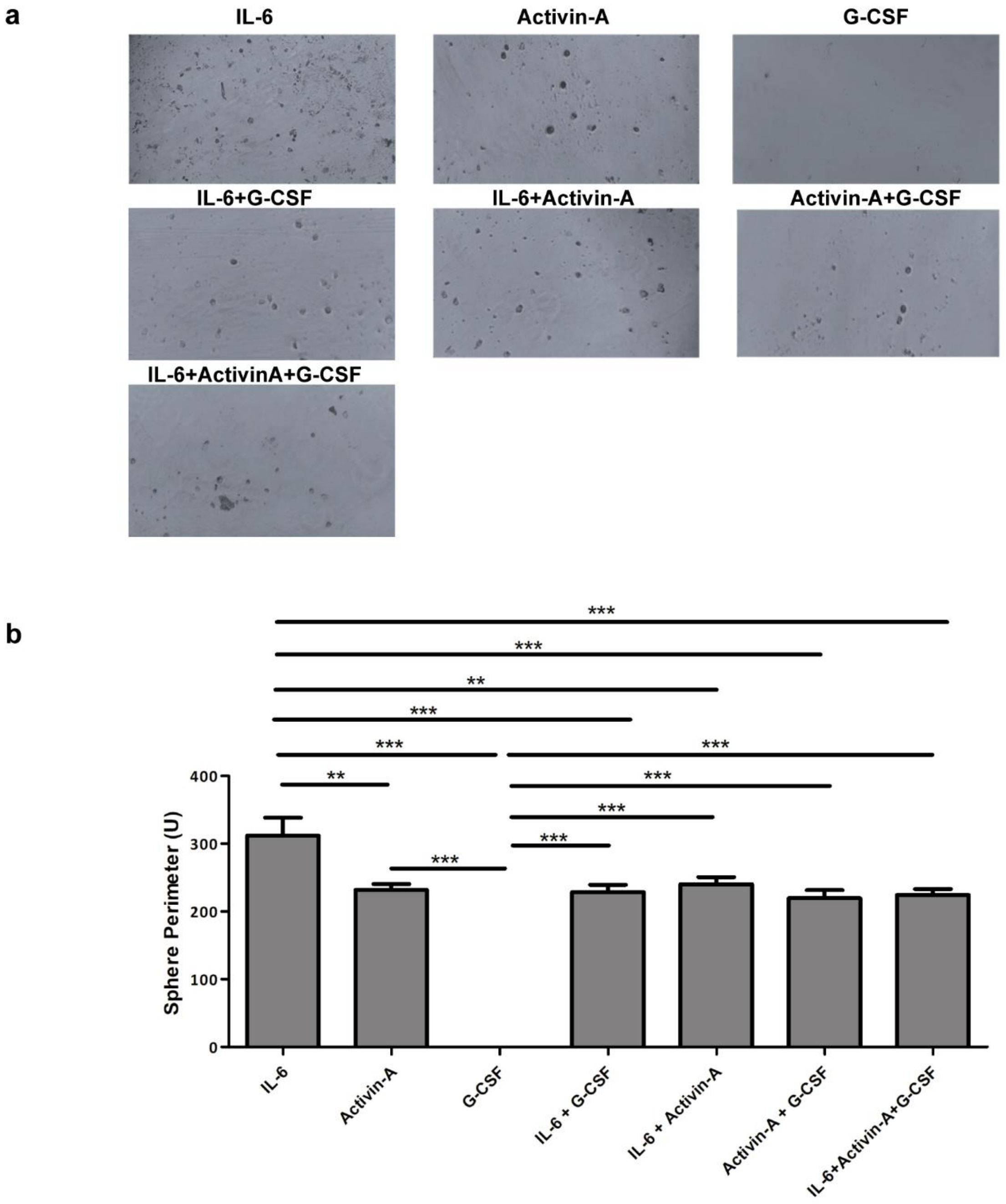

Supplementary Figure S2 – Proof-of-concept experiment illustrating cytokines-mediated dedifferentiation. (A) Sphere-forming assay performed in different conditions of cytokines' abrogation, showing that spheres were observed whenever either IL-6 or Activin-A was present. G-CSF was unable to induce sphere formation. (B) Perimeter analysis. Corroborating the previous results, bigger spheres were attained when only IL-6 was present. A magnification of 100x was used in all panels. 10 spheres were measured in each condition. Data represent means ±SEM. Differences between the means were evaluated by one-way ANOVA followed by a Bonferroni post test.

Supplementary Table S1. Statistical significance of drug-resistance assays in Figure 1F and 2F. MTX, methotrexate; Cis, cisplatin; Gem, Gemcitabine. Differences between the means were evaluated by a repetead measures ANOVA followed by a Bonferroni post test in relation to control. n.s., no significant; *, P≤0.05; **, P≤0.01; ***, P≤0.001.

	MTX (µM)			Cis (µM)			Gem (µM)		
Cell line	0.1	10	50	0.1	10	50	0.1	10	50
BEAS-2B	**	***	***	***	***	***	***	***	***
Cont1	***	***	***	***	***	***	***	***	***
RenG2	*	**	***	*	*	**	*	**	**
DRenG2	***	***	***	n.s	n.s	*	n.s	n.s	n.s
DDRenG2	**	***	***	n.s	n.s	*	n.s	n.s	n.s
SC-DRenG2	n.s	n.s	*	–	–	–	–	–	–
SC-DDRenG2	n.s	n.s	*	–	–	–	–	–	–
